# Treatment of Prolactinomas in Low-Income Countries

**DOI:** 10.1155/2015/697065

**Published:** 2015-02-09

**Authors:** Ivan Kruljac, Lora Stanka Kirigin, Mateja Strinović, Jelena Marinković, Hrvoje Ivan Pećina, Vatroslav Čerina, Darko Stipić, Milan Vrkljan

**Affiliations:** ^1^Department of Endocrinology, Diabetes and Metabolic Diseases “Mladen Sekso”, University Hospital Center “Sestre Milosrdnice”, University of Zagreb Medical School, Vinogradska Cesta 29, 10000 Zagreb, Croatia; ^2^Department of Radiology, University Hospital Center “Sestre Milosrdnice”, Vinogradska Cesta 29, 10000 Zagreb, Croatia; ^3^Department of Neurosurgery, University Hospital Center “Sestre Milosrdnice”, Vinogradska Cesta 29, 10000 Zagreb, Croatia

## Abstract

*Purpose*. In low-income countries, prolactinomas are difficult to manage with dopamine agonists (DA). We compared the effectiveness of DA in microprolactinomas as a first line treatment and as adjuvant therapy for residual macroprolactinomas treated surgically. *Methods*. Our retrospective study analyzed 78 patients, 38 with microprolactinomas and 40 with macroprolactinomas. Microprolactinomas were treated with DA. Macroprolactinomas were treated with microsurgical or endoscopic adenomectomies and adjuvant DA. Surgical remission was defined as normoprolactinemia three months postoperatively, and long-term remission as normoprolactinemia at the last control. *Results*. Surgical remission was achieved in 9 patients (23%). Postsurgical tumor mass was reduced by 50% (34–68). Residual macroprolactinoma size was greater than microprolactinoma size prior to treatment (10 mm versus 4 mm, *P* < 0.001). Both groups received similar doses of DA. Long-term remission occurred in 68% of microprolactinomas and 43% of macroprolactinomas (*P* = 0.102). Prolactin (PRL) levels at the last control were similar in both groups (23.1 versus 32.9 mcg/L, *P* = 0.347). *Conclusion*. Comparable remission rates and PRL levels were reached in microprolactinomas and macroprolactinomas using similar doses of DA. Although complete tumor resection is the goal of surgery, our study suggests that even partial surgical removal has a role in treatment of prolactinomas since it may enhance the response to DA.

## 1. Introduction

Prolactinomas represent 40% of all pituitary tumors. They are classified according to their size as macroprolactinomas (>10 mm) or microprolactinomas (<10 mm). Treatment is recommended when they are large enough to cause neurologic symptoms and when hyperprolactinemia leads to hypogonadism or galactorrhea [1]. The endocrine clinical practice guidelines recommend the use of dopamine agonists (DA) for the treatment of all symptomatic macro- and microprolactinomas [2]. The two most commonly used DA are bromocriptine and cabergoline. Cabergoline is more expensive but is more effective in normalizing prolactin (PRL) levels and in reducing tumor size. In addition, cabergoline has fewer side effects [1, 3]. Surgery is recommended in select cases when patients do not respond or are intolerant to medical therapy, severe compressive symptoms exist, cystic changes are present, or pituitary apoplexy occurs [1, 4]. However, pituitary surgery has been used as a first line treatment in patients that favored surgery over dopamine agonists with excellent results [5]. Larger tumors generally require higher doses of dopamine agonists [6, 7] and are more resistant to treatment [8]. In low income countries such as Croatia, the treatment of macroprolactinomas with medical management alone proves to be challenging. High doses of bromocriptine can lead to intolerable side effects such as nausea, postural hypotension, and dizziness [3]. Moreover, cabergoline is not registered in our country for this use, and the costs are covered from our department's annual budget. Some patients require life-long therapy and these costs are unsustainable [6]. In order to overcome this therapeutic challenge, patients harboring macroprolactinomas are first treated surgically. If additional medical therapy is required, lower doses of bromocriptine or cabergoline are used. Studies have indicated that partial surgical removal of growth hormone-secreting pituitary tumors enhances the response to somatostatin analogs in acromegaly [9, 10]. Likewise, debulking of prolactinomas may also increase the efficacy of dopamine agonists. This could potentially reduce the costs of treatment because lower doses of cabergoline could be used in patients with larger prolactinomas. Our hospital is the reference center for pituitary surgery and has been involved in pituitary microsurgery since 1986 and endoscopic surgery since 2006. We currently have two dedicated neurosurgeons who perform a minimal of 80 operations each per year with excellent results. The aim of this study was to compare the effectiveness of dopamine agonists in patients with microprolactinomas as a first line treatment and as adjuvant therapy for patients with residual macroprolactinomas.

## 2. Patients and Methods

We searched for patients with prolactinomas in our electronic database from January 1, 2010, to December 31, 2013. Microprolactinomas were defined as pituitary adenomas <10 mm in diameter with increased prolactin levels. Macroprolactinomas were defined as pituitary adenomas >10 mm in diameter along with serum prolactin levels above 100 mcg/L. Only newly diagnosed patients were included in the study. Exclusion criteria included patients who were treated with dopamine agonists prior to surgery. All patients with microprolactinomas were treated with DA. Patients with macroprolactinomas underwent microsurgical or endoscopic adenomectomies. Two skilled neurosurgeons (each performs >80 pituitary surgeries/year) operated on the patients using microsurgical or endoscopic techniques. Bromocriptine was given to patients with microprolactinomas and gradually increased to 2.5 mg/day taken as a single dose. Subsequent doses were adjusted according to PRL levels and drug tolerance. Doses were increased by 2.5 mg every 3 months when needed. If patients could not tolerate bromocriptine, cabergoline was substituted. Cabergoline was introduced at a starting dose of 0.5 mg/week and then increased by 0.5 mg increments every 3 months depending on PRL levels. DA were initiated in similar fashion in patients with macroprolactinomas who did not meet the criteria for surgical remission. Surgical remission was defined as normal PRL levels three months postoperatively. Long-term remission was defined as normal PRL levels at the last control visit. PRL was quantitatively measured using the DELFIA fluorescent method by the PerkinElmer company (normal range for women: 2.0–30.0 *μ*g/L and for men: 2.0–20.0 *μ*g/L). We used a 1.5-Tesla MRI according to the standard protocol. It included native T1- and T2-weighted imaging and dynamic T1-weighted imaging after gadolinium-base contrast medium. The longest diameter of the tumor mass was used to measure tumor size. All human and animal rights were respected during our study. The ethics committee at our institution approved this study.

### 2.1. Statistical Analyses

Patients' characteristics were assessed with descriptive statistics presented as mean and standard deviation or median with interquartile range values. Due to relatively small patient groups, independent variables were compared using Mann-Whitney test and Fisher's exact test accordingly. Changes in serum PRL levels were compared using Wilcoxon's test. Statistical analyses were performed using SAS for Windows software, version 9.1.3. (SAS Inc., Cary, NC, USA) licensed to the Zagreb University School of Medicine. *P* value <0.05 was considered significant.

## 3. Results

**Figure 1:**
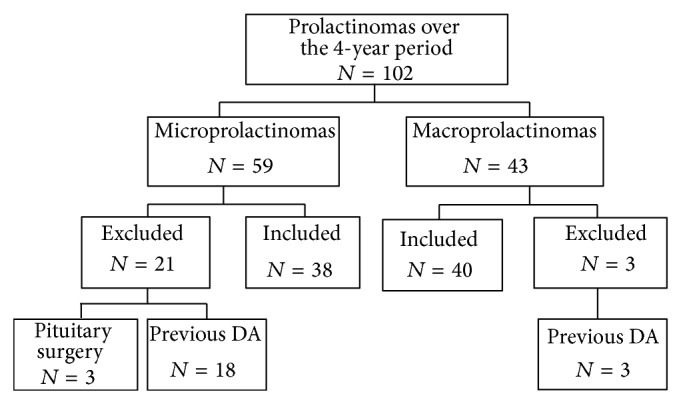
Flow of patients through the study.

**Figure 2:**
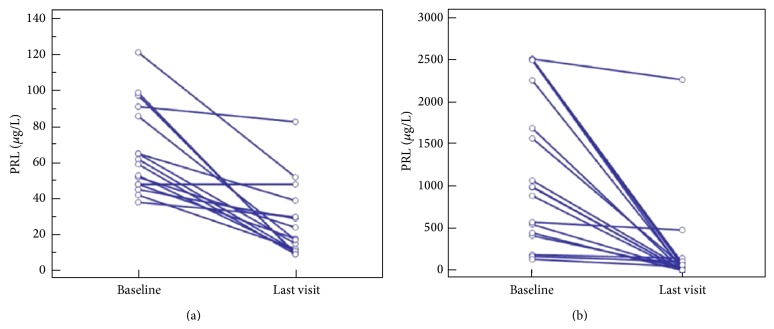
PRL levels at baseline and at the last control visit in patients with microprolactinomas (a) and macroprolactinomas (b).

We identified 102 patients with prolactinomas in our database, 59 microprolactinomas and 43 macroprolactinomas (Figure 1). Twenty-one patients with microprolactinomas were excluded from the study, 18 were previously treated with DA, and pituitary surgery was performed as first line treatment in 3 patients. Three patients with macroprolactinomas were previously treated with DA and were excluded from the study. There were 78 eligible patients with prolactinomas (55 women and 23 men); 38 had microprolactinomas with a median size of 4.0 mm (range 3.0–6.0), and 40 had macroprolactinomas with a preoperative median size of 26 mm (range 16.3–31.8). Patient and tumor characteristics are shown in Table 1. Surgical remission was achieved in 9 patients (23%). Complications occurred in three patients (8%): one patient had cerebrospinal fluid leakage, one had nasal bleeding, and one had transient diabetes insipidus. Tumor mass was reduced by a median of 50% (range 34–68) in patients with residual tumors. Patients with residual macroprolactinoma had significantly larger tumor masses than microprolactinomas (10 mm versus 4 mm, *P* < 0.001). In the macroprolactinoma group, PRL levels three months postoperatively were similar to the initial PRL levels of microprolactinomas (83.3 mcg/L (50.0–157.6) versus 63.5 (48.3–93.0), *P* = 0.275). DA were given to 23 patients with residual macroprolactinomas that did not have surgical remission. Thirty-seven percent of patients with microprolactinomas were intolerant to bromocriptine and were switched to cabergoline. Following surgery, 43% of patients with macroprolactinomas were switched to cabergoline due to intolerance. The dose of cabergoline was similar in patients with microprolactinomas (0.5 mg; range 0.50–0.69) and macroprolactinomas (0.5 mg; range 0.5–1.0), *P* = 0.123. Both groups received 2.5 mg (range 2.5–5.0) of bromocriptine (*P* = 0.853). Patients with microprolactinomas were treated with DA for a median of 25.0 months (range 15.0–87.0) and patients with residual macroprolactinomas were treated with DA for a median of 25.5 months (range 10.0–43.0), *P* = 0.404. After a median of 25.5 months, PRL levels decreased significantly in both groups (Table 2). Long-term remission was achieved in 68% of patients with microprolactinomas and 43% of patients with macroprolactinomas (*P* = 0.102). There was no statistically significant difference in terms of remission or PRL levels at the end of treatment (Figure 2). DA were discontinued in 42% (16/38) of patients with microprolactinomas and 62.5% (10/16) of these patients had relapses. Only three patients with macroprolactinomas were discontinued from medical therapy and all had relapses. Two patients with surgical remission had tumor recurrence over a 24-month period.

## 4. Discussion

Dopamine agonists are recommended as first line therapy in all symptomatic micro- and macroprolactinomas. However, in low-income countries, macroprolactinomas are difficult to manage with dopamine agonists alone due to the costs and side-effect profile of these drugs [1, 11]. Study by Verhelst et al. showed that patients with macroprolactinomas need higher median cabergoline dose to achieve normoprolactinemia, compared with those with idiopathic hyperprolactinemia or a microprolactinoma (1.0 mg/week versus 0.5 mg/week) [6]. Moreover, there is a dose-dependent suppression of serum prolactin by cabergoline in patients with prolactinomas [7]. Given the fact that macroprolactinomas tend to have higher prolactin levels, it is reasonable that macroprolactinomas require higher doses of DA. Similar studies regarding the dose effects of bromocriptine are lacking. However, similar remission rates were observed in patients with macroprolactinomas treated with 10–20 mg of bromocriptine and patients with microprolactinomas treated with 2.5–10 mg daily [11, 12], suggesting that macroprolactinomas generally require higher doses of bromocriptine. High dose bromocriptine is associated with intolerable side effects. Cabergoline is on the other hand better tolerated, but it is more expensive and most patients require life-long therapy [11]. Recommended doses of DA are 2.5 mg of bromocriptine twice daily and 0.5 mg of cabergoline twice weekly [1, 3]. In a randomized trial comparing cabergoline and bromocriptine, normoprolactinemia was achieved in 83% of patients treated with cabergoline and 59% of patients treated with bromocriptine [3]. With these optimal doses, studies have shown that, after cessation of medication, recurrences can range from 36 to 80% [13, 14]. In addition, higher recurrences are seen with larger tumors and with higher PRL levels at diagnosis [2]. One meta-analysis showed that remission after drug withdrawal was 21% for patients with microprolactinomas and 16% for patients with macroprolactinomas [15]. In our study, patients with microprolactinomas and residual macroprolactinomas were treated with similar doses of DA and had the same PRL measurements after 2 years of treatment (Figure 2). Our patients did not receive the recommended doses of dopamine agonists because high doses of bromocriptine were associated with intolerable side effects and we could not afford to give cabergoline at higher doses due to financial reasons. This could explain why our results are inferior to similar studies. Nevertheless, 68% of patients with microprolactinomas and 43% of patients with macroprolactinomas reached normoprolactinemia. Although surgery is recommended in select cases, it should be considered as an alternative to medical therapy when the costs of dopamine agonists limit their use. When surgery is performed in specialized centers, normoprolactinemia is reached in 67–88% of cases. These results are comparable to remission rates with dopamine agonists [4]. However, similar to DA withdrawal, studies have shown that 7–50% of prolactinomas that are treated surgically recur. Risks of pituitary surgery include hypopituitarism, diabetes insipidus, cerebrospinal fluid leaks, and infection [2]. Several studies have demonstrated that fewer complications are present when a more experienced pituitary surgeon performs the procedures [14]. Therefore, when surgery is considered, an experienced surgeon must be present. Due to the risks associated with these procedures, we recommend pituitary surgery as a valuable treatment option for patients with macroprolactinomas. Even partial resection has a role in treatment of prolactinomas since it may enhance the response to dopamine agonists. Microprolactinomas are well controlled with lower doses of DA, and these patients may not be subjected to the risks of surgery.

## 5. Conclusion

Pituitary surgery may be a valuable first line treatment of macroprolactinomas. Comparable remission rates and prolactin levels were reached in microprolactinomas and residual macroprolactinomas using similar doses of dopamine agonists. Although the complete resection of the tumor mass is the goal of surgery, our study suggests that even partial surgical removal has a role in treatment of prolactinomas since it may enhance the response to dopamine agonists.

## Figures and Tables

**Table 1:** Characteristics of men and women with micro- and macroprolactinomas at diagnosis.

	Total	Microprolactinomas	Macroprolactinomas	*P*
Number (%)	78 (100)	38 (54.3)	40 (45.7)	NA
Pretreatment size (mm)	6.5 (4.0–18.0)	4.0 (3.0–6.0)	26.0 (16.3–31.8)	<0.001
Men number (%)	23 (100)	0 (0)	23 (100)	NA
Women number (%)	55 (100)	38 (69.1)	17 (30.9)	NA
Age (years)	40.0 (32.0–53.0)	36.5 (32.000–50.0)	43.0 (32.0–62.0)	0.259
Serum prolactin levels (mcg/L)	113.6 (60.5–941.5)	63.5 (48.3–93.0)	995.5 (417.0–2500.0)	<0.001

NA: not analyzed.

**Table 2:** Treatment details for microprolactinomas treated with dopamine agonists and macroprolactinomas treated with adjuvant medical therapy following surgery. Remission and relapse rates are presented in the two groups.

	Microprolactinomas	Macroprolactinomas	*P*
Bromocriptine dose (mg)	24/38 2.5 (2.5–5.0)	13/23 2.5 (2.5–5.0)	0.596 0.853
Cabergoline dose (mg)	14/38 0.5 (0.5–0.69)	10/23 0.5 (0.5–1.0)	0.596 0.123
Surgical remission	—	23% (9/40)	
Duration of treatment (months)	25.0 (15.0–87.0)	25.5 (10.0–43.0)	0.404
Long-term remission	68% (26/38)	43% (10/23)	0.122
PRL at the last control	23.1 (12.4–35.8)	32.9 (12.0–70.0)	0.279
Withdrawal of therapy	16/38	3/23	0.022
Normal PRL after withdrawal	15.8% (6/38)	0% (0/3)	0.073
